# An Alginate/Cyclodextrin Spray Drying Matrix to Improve *Shelf Life* and Antioxidant Efficiency of a Blood Orange By-Product Extract Rich in Polyphenols: MMPs Inhibition and Antiglycation Activity in Dysmetabolic Diseases

**DOI:** 10.1155/2017/2867630

**Published:** 2017-11-02

**Authors:** Maria Rosaria Lauro, Lucia Crascì, Virgilio Giannone, Gabriele Ballistreri, Simona Fabroni, Francesca Sansone, Paolo Rapisarda, Anna Maria Panico, Giovanni Puglisi

**Affiliations:** ^1^Department of Pharmacy, University of Salerno, Via Giovanni Paolo II, 84084 Fisciano, Italy; ^2^Department of Drug Science, University of Catania, Viale A. Doria, 95100 Catania, Italy; ^3^Department of Agricultural and Forest Sciences, University of Palermo, Viale delle Scienze Ed.4, 90128 Palermo, Italy; ^4^Consiglio per la Ricerca in Agricoltura e l'Analisi dell'Economia Agraria (CREA) – Centro di ricerca Olivicoltura, Frutticoltura e Agrumicoltura, Corso Savoia, 190, 95024 Acireale, Italy

## Abstract

Alginate and *β*-cyclodextrin were used to produce easily dosable and spray-dried microsystems of a dried blood orange extract with antidysmetabolic properties, obtained from a by-product fluid extract. The spray-dried applied conditions were able to obtain a concentrate dried extract without the loss of AOA and with TPC and TMA values of 35–40% higher than that of the starting material. They were also effective in producing microparticles with 80–100% of encapsulation efficiency. The 2% sodium alginate was capable of improving the extract *shelf life*, while the beta-cyclodextrin (1 : 1 molar ratio with dried extract) prolonged the extract antioxidant efficiency by 6 hours. The good inhibition effect of the dried extract on the AGE formation and the MMP-2 and MMP-9 activity is presumably due to a synergic effect exerted by both anthocyanin and bioflavonoid extract compounds and was improved by the use of alginate and cyclodextrin.

## 1. Introduction

The term “food by-products” indicates that “food wastes” are ultimate substrates for the recovery of functional compounds, as phytochemicals, to develop new products with additional value [1]. Blood orange processing wastes still contain large amounts of anthocyanins, bioflavonoids, and other polyphenols [2]. Many of these compounds are known for their antioxidant effect depending on their concentration [3–5] and their ability to inhibit the metalloproteinase (MMP) activity [6] as MMP-2 and MMP-9, overexpressed during dysmetabolic diseases. In addition, a recent study reported the ability of dietary polyphenols to lower the advanced glycation end product (AGE) formation and protein glycation [7]. For these reasons, phytochemicals may be considered potential bioactive additives for functional food to prevent dysmetabolic pathologies. So, they could be conveniently recovered for nutraceutical purposes.

Our focus was on a Sicilian blood (pigmented or red) orange (cv. “Moro,” “Tarocco,” and “Sanguinello”) by-product fluid extract (ExF) potentially active on dyslipidemic pathologies [8, 9].

Unfortunately, the fluid extracts are difficult to handle from industry and are more unstable with respect to a dried product. In fact, phytochemical content easily oxidizes/degrades [10]. To overcome this problem, a spray-dried extract (ExMR) was produced. The spray drying technique is able to prevent the appreciable losses of the extract's bioactive compounds without altering the health potential of the extract [11]. To protect phytochemicals from oxidation/degradation phenomena and enhance extract bioavailability, shelf life, and antioxidant efficiency, bioactive ExMR microparticles easily added to common foods of daily diet to enhance the patient compliance were obtained. As carriers, edible biocompatible films and coatings present in the GRAS list and able to deliver several vitamins, antioxidants, and probiotics in food systems [12] were chosen.

In particular, sodium alginate (ALG) is a water-soluble polymer capable of forming a hydrogel polymer matrix which allows a good diffusion of the bioactive compounds. Moreover, several studies showed that treatments with sodium alginate mitigate the glucose excursions, reduce insulin responses, increase satiety, and decrease energy intake and obesity [13, 14]. For its activities, ALG can be used in synergy with the extract in dyslipidemic diseases such as obesity, diabetes, and hypercholesterolemia. Instead, *β*-cyclodextrin (CD) and its derivatives are able to improve the physicochemical properties of the guest molecules [15], such as degradation and solubility [16], also masking their bitter taste [17].

In our study, solubility, solid state, dissolution properties, and *shelf life* of microparticle-loaded extract were studied and compared to those of the fluid extract to evaluate the effectiveness of the used spray drying technique. The influence of parameters such as the polymer concentration and the extract/polymer ratio on particle yield, distribution, and morphology was also investigated. Furthermore, the extract protection efficacy of the used selected coated polymers has been evaluated. Considering the synergistic involvement of free radicals, AGEs and MMPs on the onset of dysmetabolic diseases [18], the antioxidant activity (ORAC assay), the antiglycation activity, and the inhibition capacity on MMP-2 and MMP-9 (gelatinases involved in vascular remodeling with a consequence of high levels of cholesterol and triglycerides) [6, 19] of both formulated and unformulated extracts have been evaluated.

## 2. Materials and Methods

### 2.1. Materials

The fluid aqueous extract obtained from blood orange processing wastes (ExF) was produced by Ortogel SpA (Caltagirone, Sicily, Italy). Beta-cyclodextrin was supplied by Roquette Frères (Lestrem, France). Sodium alginate (ALG), fluorescein (FL), AAPH (2,2′ azobis(2-methylpropionamide) dihydrochloride) 97%, Trolox (6-hydroxy-2,5,7,8-tetramethylchroman-2-carboxylic acid) and HEPES (4-(2-hydroxyethyl)piperazine-1-ethanesulfonic acid), aminoguanidine bicarbonate 97% (AMG), bovine serum albumin (BSA), D-(−)-fructose, and sodium azide (NaN_3_) were purchased from Sigma-Aldrich Srl (Milan, Italy). Anthocyanins (cyanidin-3-glucoside, cyanidin-3,5-diglucoside, cyanidin-3-rutinoside, and cyanidin-3-sophoroside;delphinidin-3-glucoside and delphinidin-3,5-diglucoside; pelargonidin-3-glucoside and pexlargonidin-3,5-diglucoside; peonidin-3-glucoside; and malvidin-3-glucoside) and flavanones (hesperidin, narirutin, and didymin) were purchased from Extrasynthèse (Genay, France). OmniMMP fluorescent substrate Mca-Pro-Leu-Gly-Leu-Dpa-Ala-Arg-NH_2_, MMP-9 (refolded) (human) (recombinant) (catalytic domain) and MMP-2 (catalytic domain) (human) (recombinant) were purchased from Vinci-Biochem Srl (Firenze, Italy). Solvents for chromatography were HPLC grade (Merck KGaA, Darmstadt, Germany). All the other chemicals used in the study were of analytical grade and were obtained commercially.

### 2.2. Manufacture of Spray-Dried ExMR Product

A Buchi Mini Spray Dryer B-191 (Buchi Laboratoriums-Tecnik, Flawil, Switzerland) was used for the drying process: inlet temperature, 120°C; outlet temperature, 68–71°C; spray flow feed rate, 5 ml/min; nozzle diameter, 0.7 mm; drying air flow, 500 l/h; air pressure, 6 atm; and 100% aspirator.

### 2.3. Qualitative and Quantitative Analyses of Bioactive Compounds in Fluid (ExF) and Spray-Dried Aqueous Extract (ExMR)

#### 2.3.1. Qualitative Analysis

*Anthocyanin Purification and HPLC-PDA-ESI/MSn Analysis of Anthocyanins*. The extracts were diluted in water for HPLC analysis of anthocyanins. Aqueous extracts were loaded onto C18 Bond Elut SPE cartridges (Varian Inc., Palo Alto, CA, USA) that were previously conditioned with methanol and pure water. The anthocyanins were adsorbed by these cartridges while other soluble compounds such as sugars and acids were removed by washing the columns with pure water. Anthocyanins were eluted with methanol containing 1% formic acid. The acidified methanol solutions were evaporated to dryness; then, the dried fractions were redissolved in 7% aqueous formic acid. Then, the samples were filtered through a 0.45 *μ*m membrane filter (Albet, Barcelona, Spain) and injected into the HPLC-MS*^n^* chromatographic system to identify the individual anthocyanins.

Evaluation of anthocyanins was performed on a Chromolith Performance RP-18 endcapped column (100 × 3.0 mm i.d., monolithic particle size; Merck KGaA, Darmstadt, Germany) using an ultrafast HPLC system coupled to a photodiode array (PDA) detector and Finnigan LXQ ion trap equipped with an electrospray ionization (ESI) interface in series configuration (Thermo Electron, San Jose, CA, USA). HPLC conditions and MS parameters were reported as the same in a previous work [20]. Anthocyanins were identified by using their retention time (tR), MS, and MS*^n^* spectral data in a positive ion mode. In addition, comparison of the MS spectral data with those of pure standards and/or those reported in literature was performed. The relative compositions (%) of the individual anthocyanins were calculated from the peak areas of the chromatogram detected at 520 nm, using Xcalibur versus 2.0.7 software (Thermo Electron).

*HPLC-PDA-ESI/MSn Analysis of Flavanones*. Flavanone glycosides, expressed as hesperidin (Hd) equivalents (g/100 g of extract), were determined by HPLC [21] using the HPLC-PDA-ESI/MS*^n^* equipment described above. A sample of the extract was dissolved in dimethyl sulfoxide and diluted with the mobile phase, filtered through a 0.45 *μ*m membrane filter (Albet, Barcelona, Spain), and then injected directly into the column. The eluent was water : acetonitrile : acetic acid (79.5 : 20 : 0.5), and the flow rate was 800 *μ*l/min. The individual flavanones were detected at 280 nm. MS parameters were the same as those described for anthocyanin analysis. Flavanones were identified by using their retention time (tR), MS, and MS*^n^* spectral data in a negative ion mode and also by comparison of the MS data with those of pure standards and/or those reported in literature.

#### 2.3.2. Quantitative Analysis

*Total Polyphenol Content (TPC)*. The polyphenol contents of ExF and ExMR were determined by the Folin-Ciocalteau method according to Aiyegoro and Okoh [22], with slight modifications. 2 mg/ml of both extracts was dissolved in distilled water. Then, 2.5 ml of 10% Folin-Ciocalteau reagent and 2 ml of Na_2_CO_3_ (2% *w*/*v*) were added to 0.5 ml of each samples. The resulting mixtures were incubated at 45°C with shaking for 15 min. The absorbance of the samples was measured at 765 nm using UV/visible light. The standard curve (*y* = 4.292−0.0293*x*; *R*^2^ = 0.9951) was prepared by 0, 0.05, 0.1, 0.15, 0.2, and 0.25 mg·ml^−1^ solutions of gallic acid in water. TPC values are expressed as milligram of gallic acid equivalents (GAE)/g of extract.

*Total Monomeric Anthocyanin (TMA) Content*. TMA content was assayed by the pH differential method [23] by using a UV/Vis spectrophotometer (Varian Cary 100 Scan, Palo Alto, CA, USA). Total anthocyanin content was expressed as g of cyanidin-3-glucoside equivalents (C3G)/100 g of extract on a dry weight (DW) basis.

### 2.4. Solubility Studies

#### 2.4.1. ExMR Solubility

Solubility test of dried ExMR was performed in distilled water (3.0 ± 0.2 g/l) and in gastric (GF; pH 1.2) (3.5 ± 0.1 g/l) and intestinal (IF; pH 7.5) (3.8 ± 0.2 g/l) simulated fluids, without enzymes (USP 37) by UV/Vis spectrometry at *λ* = 310 nm and expressed as hesperidin equivalents (Hd). Each analysis was made in triplicate.

#### 2.4.2. Phase Solubility

2.5 × 10^3^ mol of ExMR, expressed as Hd, was suspended in 100 ml of water. Different amounts [24] of CD (1 : 3, 1 : 2, 1 : 1.5, 1 : 1, and 2 : 1 ExMR/CD molar ratio) were added. The samples were shaken, stored at 25°C for 1 hour, and then centrifuged (5 min at 3000 rpm). The supernatants were analyzed in UV apparatus (1 cm cell; *λ* = 278 nm).

### 2.5. Microparticle Preparation

ExMR was suspended (3 : 1 polymer : extract weight ratio) in 1% or 2% (*w*/*v*) ALG aqueous solution, under magnetic stirring [25], to give the ExMR1 (1% ALG) and ExMR2 (2% ALG) microsystems. In the second step, CD (1 : 1 CD : ExMR molar ratio) was dissolved in 2% *w*/*v* ALG water solution (3 : 1 ALG : ExMR weight ratio), to obtain ALGCDExMR microparticles. ALG-free microparticles (CDExMR) were used as control. The spray drying conditions were reported as the same in ExMR preparation. All the spray-dried microparticles were carried out in triplicate, collected, and stored under vacuum (48 h at room temperature).

### 2.6. Microparticle Properties and Characterization

#### 2.6.1. Particle Size Analyses

Isopropanol was used as a suspending agent for all samples. A Beckman Counter LS 230, Particle Volume Module Plus, UK (instrument obscuration: 8–12%), was used to examine the particle size in triplicate applying the Fraunhofer model. The results were expressed as the median diameter of the particles (d50).

#### 2.6.2. Morphology

The microphotographs of the morphology of all samples were acquired by a confocal (Leica TCS SP2, CF) and a fluorescent microscope (Zeiss Axiophot, FM).

All images were equipped with 63 × 1.4 NA plan apochromat oil immersion objectives (Carl Zeiss Vision, München-Hallbergmoos, Germany) and standard DAPI (4′,6-diamidino-2-phenylindole) optics that adsorb violet radiation (max 372 nm) and emit blue fluorescence (max 456 nm).

#### 2.6.3. Yield of the Process (*Y*), Encapsulation Efficiency (EE), and Extract Content in Microsystems

*Y* was gravimetrically determined and expressed as the weight percentage of the final product compared to the total amount of the sprayed materials.

EE was calculated according to Sansone et al. [10].

(1) $$\begin{array}{c} EE\;\left(\%\right)=\frac{actual\;extract\;content}{theoretical\;extract\;content}. \end{array}$$

The actual extract content was calculated as Hd concentration used as a marker and determined in the supernatant solutions of 15 mg of microsystems dissolved in MeOH (15 ml; sonicated for 5 min, centrifuged for 10 min at 300 rpm) by HPLC Agilent equipment (Agilent 1100 series system; model G-1312 pump; Rheodyne Model G-1322A loop (20 *μ*l); DAD G-1315 detector; 150 × 3.9 mm i.d. C18 *μ*-Bondapack column). The flow rate is 1.0 ml·min^−1^. The following are the mobile phases: water (solvent A) and methanol (solvent B). The elution gradient was shown as follows: 0 → 5 min (15 → 30%) B, 5 → 10 min (30 → 35%) B, 10 → 20 min (35 → 50%) B, 20 → 30 min (50 → 75%) B, 30 → 35 min (75 → 95%) B, and 35 → 40 min (100%) B. A DAD detector was set at *λ* = 283 nm. Hd reference standard solutions were prepared at five concentration levels in the range 1–40 *μ*g/ml. Linear least squares regression equation was derived from the peak area corresponding to Hd (*y* = 1798.3*x*−54.938, *R* = 0.9996), where *y* is the peak area and *x* the used concentration.

#### 2.6.4. Differential Scanning Calorimetry (DSC) and Fourier-Transform Infrared Spectroscopy (FTIR)

*DSC*. An indium-calibrated Mettler Toledo DSC822e (OH, USA) was used exposing all the samples to two thermal cycles: a dehydration cycle up to 130°C (heating rate of 20°C/min; temperature maintained at 130°C for 15 min in order to remove the residual solvent); afterwards, the samples were cooled at 25°C and heated up to 350°C (heating rate of 10°/min) [6]. The analyses were carried out in triplicate.

*FTIR*. A Jasco FT-300 (Tokyo, Japan) Fourier-transform IR spectrometer was used to analyze all samples in two steps: first, the material was dried in a vacuum oven to reduce the presence of water and then analyzed as KBr discs in the spectral region 650–4,000 cm^−1^ at a resolution of 8 cm^−1^.

### 2.7. Stability Studies

#### 2.7.1. Accelerated Stability

The stability test was performed according to accelerated stability studies reported in the ICH guidelines (International Conference on Harmonization of Technical Requirements of Pharmaceutical for Human Use, 2003) in a climatic and thermostatic chamber (Mod.CCP37, AMT srl, Milan, Italy), at 40°C ± 2°C/75% RH ± 5% RH for one week and then analyzed by UV and HPLC in terms of extract content and TPC variation. Chromatographic peaks were identified on the basis of the retention times and confirmed by coinjections with an internal standard [14].

#### 2.7.2. Functional Stability (Oxygen Radical Absorbance Capacity: ORAC Assay)

In order to determine the *in vitro* antioxidant capacities of both ExF and ExMR, the ORAC method [26] was employed. Also, a comparison between ORAC unprocessed (ExMR) and processed extract by ICH (ExMR ICH) was performed.

The fluorescence probe fluorescein (FL, 10 nM) was used as a reference compound attacked from peroxyl free radicals that are generated from APPH (100 mM) solution. In order to calculate the area under a curve (AUC) of the tested compounds (12.5 *μ*g/ml), the reaction was following at 37°C (pH 7.0) until a fluorescence decay of FL solution in the presence of APPH. Each measurement was repeated at least three times, using a Wallac 1420 Victor 96-well plate reader (PerkinElmer, USA) with a fluorescence filter (excitation 485 nm, emission 520 nm). The Trolox (12.5 *μ*M) was used as an antioxidant control.

The ORAC value refers to the net protection area under the quenching curve of fluorescein in the presence of an antioxidant. The final results (ORAC value) were calculated and expressed in ORAC units (Trolox micromol per microgram of sample (*μ*mol/*μ*g)).

(2) $$\begin{array}{c} ORAC\;value\left(\mu mol/\mu gram\right)=\frac{K\left(S\;sample–S\;blank\right)}{\left(S\;Trolox–S\;blank\right)}, \end{array}$$

where *K* is a sample dilution factor and *S* is the area under the fluorescence decay curve of the sample, Trolox or blank, calculated with Origin®7 (OriginLab Corporation, Northampton, USA).

### 2.8. In Vitro Dissolution/Release Tests

ExMR (100 mg, sink conditions) or produced formulation corresponding to the same amount of pure dried extract was carried out under sink conditions (corresponding to about) in water using a SOTAX AT smart apparatus (Basel, CH), on a line with a spectrophotometer at *λ* = 310 (UV/Vis spectrometer Lambda 25, PerkinElmer Instruments, MA, USA), and USP 37 dissolution test apparatus n.2: paddle, 100 rpm at 37°C. All the dissolution/release tests were made in triplicate; only the mean values are reported in a graph (standard deviations < 5%).

### 2.9. Antioxidant Efficiency

**Figure 1:**
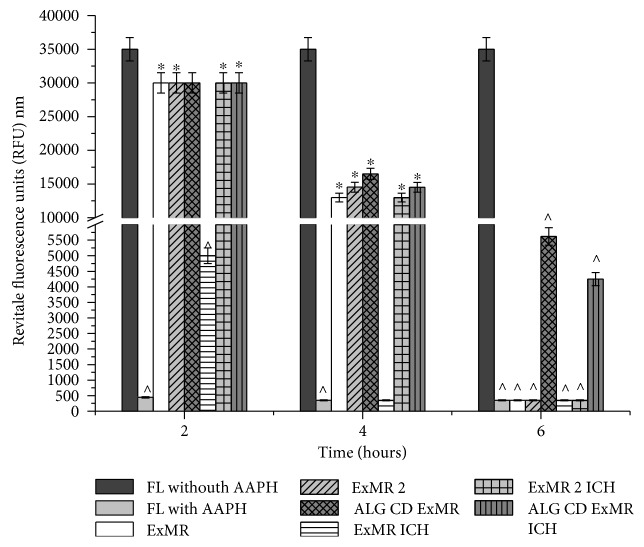
Antioxidant efficiency of unformulated and formulated extracts. Data represent the mean of three independent experiments ± SD. ^∗^*p* < 0.05 or ^^^*p* < 0.01 compared with FL without AAPH. ICH: samples processed according to ICH guidelines.

To determine the antioxidant efficiency of formulated and unformulated samples, a modified ORAC assay was used [6]. Briefly, both 25 *μ*l extracts of all samples were placed in 96-well tissue culture plates. 100 *μ*l FL (10 nM) solution was added to each well to initiate the assay. Then, 25 *μ*l AAPH (100 mM) solution was added to all wells, except for the negative control, to which 25 *μ*l phosphate buffer solution was added. A FL solution without AAPH was used as negative control. A timer was started upon introduction of the free radical generator, and the plate was stored in the dark at 37°C. Unlike the previous method (reported in paragraph 2.7.2.), at each specified time point, the fluorescence of the solution was measured (excitation 492 nm, emission 535 nm) and plotted as a function of time [27, 28], using Origin7 (OriginLab Corporation, Northampton, USA). The *y*-axis graphs in Figure 1 were split from 6000 to 10000 RFU.

### 2.10. Antiglycation Activity

According to the method of Derbré et al. [29] with slight modifications, we evaluated the inhibition of fluorescence produced by AGE formation through Maillard reaction. Briefly, as optimum AGE formation, the protein model bovine serum albumin (BSA) (10 mg/ml) was incubated with D-fructose (0.5 M) in phosphate buffer 50 mM pH 7.4 (NaN_3_ 0.02%) to obtain positive controls. BSA alone was the negative control corresponding to no fluorescence AGE formation. The aminoguanidine (AMG) (400 *μ*g/ml) was used as reference compounds for its AGE inhibition property [30]. The final glycated BSA solutions (300 *μ*L) alone and with the sample (400 *μ*g/mL) were incubated at 37°C in a 96-well microtiter closed with their silicon lids for 7 days. The AGE fluorescence measurement (*λ*exc 370 nm; *λ*em 440 nm) is performed using a VICTOR Wallac 1420 Multilabel Counter fluorimeter (PerkinElmer, USA). The results are reported in relative fluorescence units (RFU), and the percentage of inhibition with respect to the positive control (BSA with fructose) is calculated from the following equation:

(3) $$\begin{array}{c} \%\;of\;inhibition=\left\{1-\frac{RFU\;sample\left(nm\right)}{RFU‐positive\;control\left(nm\right)}\right\}\times 100. \end{array}$$

### 2.11. Inhibitory Activity on MMP-2 and MMP-9

The MMP inhibition assay of unformulated and formulated ExMR was based on the inhibition of the hydrolysis of the fluorescence-quenched peptide substrate Mca-Pro-Leu-Gly-Leu-Dpa-Ala-Arg-NH_2_ (Vinci-Biochem Srl). The assay was performed according to Crascì et al. [31]. The results were plotted with Origin7 (Originlab Corporation, Northampton, USA) software and are expressed as concentration of inhibitors that reduced of 50% the MMPs activity (IC_50_).

## 3. Results and Discussion

### 3.1. Characterization and Stability of Unprocessed (ExF) and Processed (ExMR) Extracts

**Figure 2:**
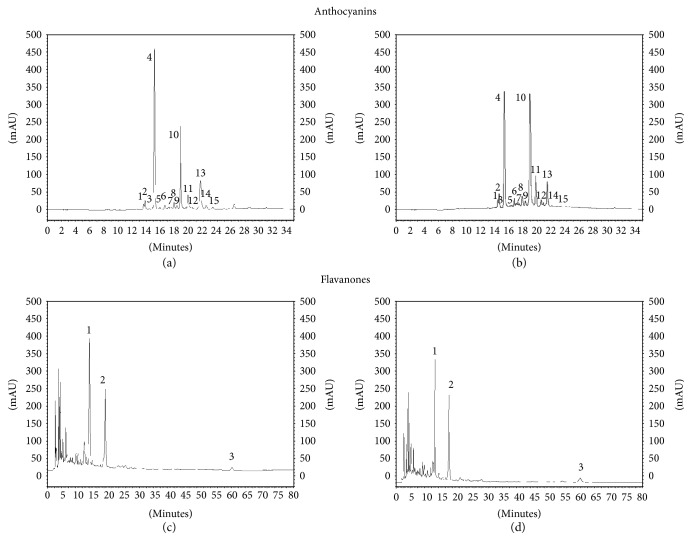
HPLC chromatograms of anthocyanins of ExMR (a) and ExF (b) detected at 520 nm (see Table 1). HPLC chromatograms of flavanones of ExMR (c) and ExF (d) detected at 280 nm (see Table 2).

Both the extracts (ExF and ExMR) were analyzed by HPLC-MS and showed four major eluted constituents (Tables 1 and 2), two anthocyanins, cyanidin 3-(6″-malonyl) glucoside (CMG, *t*_R_ 18.9 min) and cyanidin 3-glucoside (C3G, *t*_R_ 15.2 min) and two *O*-glycosylated flavonoids, hesperidin (Hd, *t*_R_ 18.5 min) and narirutin (Nr, *t*_R_ 13.5 min) (Figure 2).

In agreement with previous data reported in literature [32, 33], the major component Hd was chosen as marker [6]. Both Hd and C3G, as bioflavonoid and anthocyanin markers, respectively, were used as comparison compounds to determine the bioactive content in MMP studies.

The analytic data of ExF showed that the TPC (Table 3) and TMA values were equal to 35% and 40% of the spray-dried ExMR extract, respectively. In fact, in ExF, the levels of TPC (mg gallic acid/g extract) and TMA (%) were 12.0 ± 0.18 mg/g and 0.322 ± 0.02%, respectively. The antioxidant activity (Table 3) expressed in ORAC units (AOA) was 1.9 ± 0.8 ORAC units. The evaluation of the extract properties is essential to obtain spray-dried powders with optimized physicochemical and biological properties.

### 3.2. Spray Drying Process

ExF has been spray dried to obtain a powder extract which can be easily handled. The spray drying technique is the most commonly used in food and nutrapharmaceutical industries [34]. It is a crucial step that can affect the extract stability [35] and plays an important role in determining the properties and cost of dried products [36]. On this consideration, it is interesting to note that the selected spray dryer conditions were able to obtain a product yield equal to 60% and without loss of AOA activity (2.6 ± 0.3 ORAC units). Furthermore, the TPC and TMA values (33.5 ± 0.21 mg/g and 0.814 ± 0.1%, resp.) were higher than that of ExF. These results are probably due to the spray drying parameters such as the inlet temperature (120°C) which were optimized in order to protect and reduce the loss of polyphenols because of degradation [25].

### 3.3. Solubility Studies

ExMR has a slight water solubility (3.0 ± 0.1 mg/l) at room temperature that was notably affected by the presence of CD used as enhancer of dissolution rate, limiting factor of *in vivo* bioavailability.

The amount of CD required to increase a sample bioavailability can be evaluated by the phase solubility studies and can affect the various processes occurring during the delivery in the gastrointestinal (GI) environment [37].

**Figure 3:**
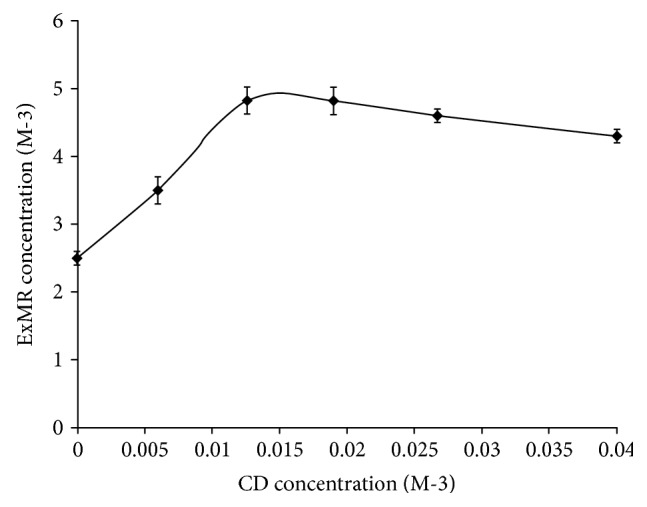
Solubility phase diagram of ExMR in the presence of *β*-cyclodextrin.

The phase solubility (Figure 3) showed a Bs-type profile [24]. CD is capable to enhance the ExMR solubility with a linear increase below the 1 : 2 ExMR/CD molar ratio. The ascending portion indicates a 1 : 1 stoichiometry complex; at higher CD concentrations appears a short plateau indicating the formation of an insoluble or with a different stoichiometry complex in the solution. For this reason, conventionally, we assumed a 1 : 1 ExMR molar ratio to obtain an improvement of water solubility (from 3.0 ± 0.1 mg/l to 6.0 ± 0.5 mg/l) and an enhancement of the dissolution rate of the extract, as confirmed by the dissolution/release test.

### 3.4. Spray-Dried Microparticle Properties and Characterization

#### 3.4.1. Alginate Microparticles (ExMR1 and ExMR2)

**Figure 4:**
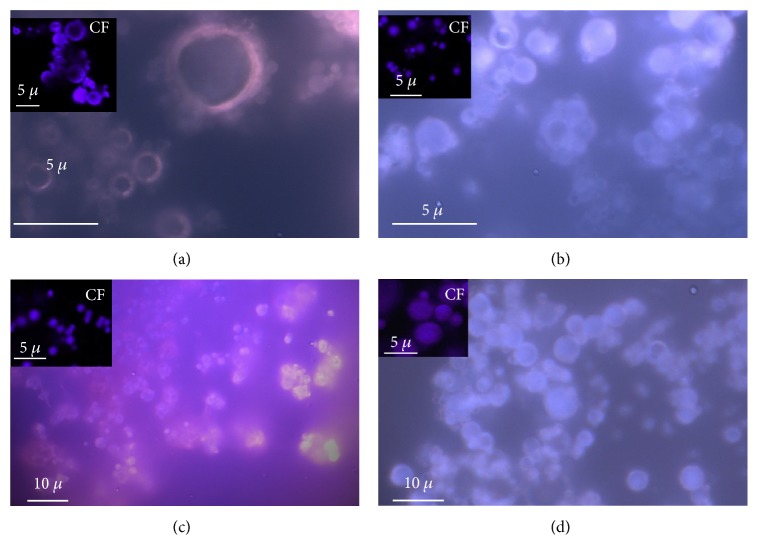
Fluorescence (FM, ×63 and ×40) and confocal (CF) microphotographs of ExMR1 (a), ExMR2 (b) CDExMR (c), and ALGCDExMR (d) microsystems.

In the first step, in order to achieve stable microsystems, ExMR was spray dried using 1% or 2% ALG water solutions *w*/*v* as coating polymer and 3 : 1 polymer : extract weight ratios. The best formulation was in the presence of 2% of ALG (ExMR2). The low amount (1%) of ALG in ExMR1 produced microparticles with greater particle size (5 ± 0.8 *μ*m) than ExMR2 (1.5 ± 0.5 *μ*m), probably due to aggregate formation and high moisture content [38, 39] (Figure 4(a)). For this reason, 2% of ALG formulation (ExMR2) were selected. The results showed that the selected parameters (spray drying conditions and 2% of ALG) were able to produce well-formed microparticles (Figure 4(b)) and to obtain a good EE (80.0%). These were due to the ALG amount, able to reduce in solid dispersion the molecular mobility of the bioactive compounds avoiding the phase separation, while the spray drying parameters were effective to lower the accumulation on the chamber wall [24, 40–43].

**Figure 5:**
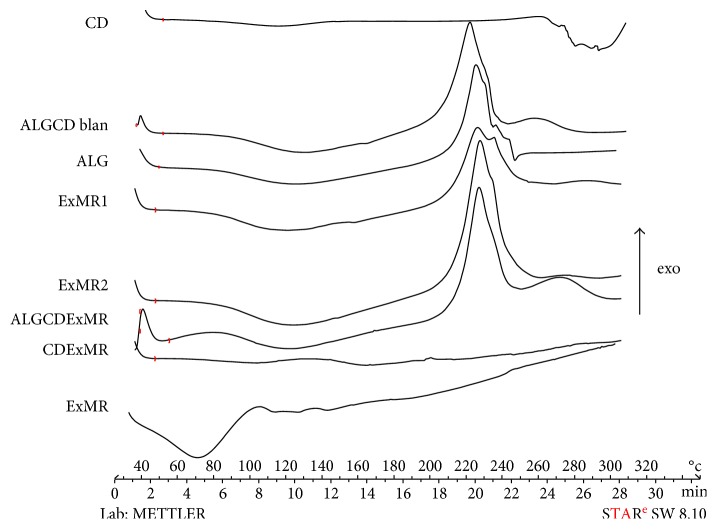
Differential scanning calorimetry of pure materials (ExMR, CD, and ALG), micropowders, blank (ALGCD blank), and loading microsystems (ExMR1, ExMR2, CDExMR, and ALGCDExMR).

The ExMR2 DSC thermogram (Figure 5) did not show any extract peak, confirming its complete encapsulation, such as that supported by FTIR analysis. Moreover, ALG shows high decomposition temperature. In fact, the ALG thermogram exhibits a first endothermic peak at 100°C (correlated to the release of water) and an exothermic peak at 250°C, due to pyrolysis reaction in the polymer [44]. Since, some food manufacturing processes, especially baked good production, required high cooking temperature (150°–200°C) [45]. The ALG thermal behavior of the produced formulation could be suitable to protect the loaded active ingredient also to prevent the premature crystallization of the sugars [46] present in the extract.

**Figure 6:**
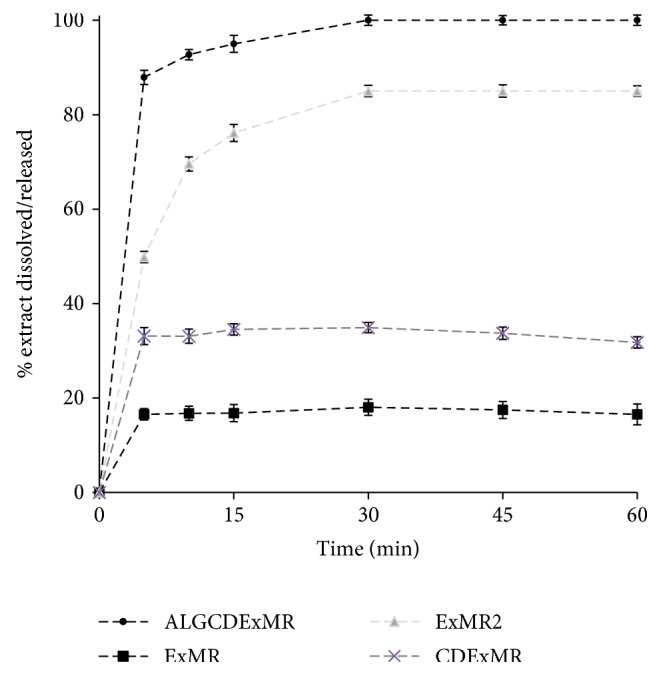
Dissolution/release profile of ExMR2, CDExMR, and ALGCDExMR in comparison with ExMR micropowder dissolution profiles in water.

2% ALG water solution is also able to improve the dissolution rate of ExMR (Figure 6). In fact, about 49% of the extract were released from ExMR2 in 5 min with respect to about 16% of pure extract that dissolved at the same time. This behavior was probably due to the presence of ALG that increase the extract-water interaction due to its high hydrophilic behavior [6, 13]. Because extract-water release was incomplete (Figure 6) (85% in 30 min), in the second step, microparticles with enhancement of the dissolution rate were developed in the presence of CD.

#### 3.4.2. Alginate/Cyclodextrin Microparticles (CDExMR and ALGCDExMR)

The presence of CD reduces the particle size during the spray drying process and improves the EE (90.0–100.0%). In fact, the micrograph of the batches CDExMR and ALGCDExMR showed the presence of small microparticles (Figures 4(c) and 4(d)) with dimensions of about 0.5 ± 0.02 and 1.0 ± 0.2 *μ*m, respectively.

The CDExMR thermogram shows a series of peaks from 180°C to 280°C which are superimposable with that of pure ExMR, with slight shifts. This behavior confirms that the 1 : 1 CD/ExMR molar ratio is unable to form a complex. Instead, the shifts show that microparticle components interact only to form −H bonds, such as confirmed by FTIR spectroscopy (amplification of OH band at 3500 cm^−1^; data not shown) [47].

The −H bonds between CD and ExMR could be responsible of the enhancement of the extract dissolution rate. About 33.0% of the extract dissolved from CDExMR in water after 5 minutes, with respect to about 16.0% of pure ExMR that dissolved at same time. Moreover, when used together, ALG and CD act synergistically enhancing the dissolution/release of ExMR (about 90% after 5 min) from ALGCDExMR, and all loaded doses were released (100% in 30 min).

### 3.5. Accelerated and Functional Stability (ICH Guidelines) and ORAC Test

The extract powders obtained from blood orange processing wastes are rich in polyphenols, easily subject to oxidation/degradation phenomena. This behavior is a critical point for their use in food or pharmaceutical field [48]. To examine the *shelf life*, the antioxidant efficiency and the effect of the ALG and CD polymers, and the microencapsulation processes on the stability of the extract in storage conditions, the “real-time” stability was reproduced according to ICH guidelines in a climatic chamber and in a brief time period in extreme conditions. The functional stability of ICH extract was performed by the ORAC assay.

After one week at 40°C, an increase in ExMR weight, determined by the gravimetric method, was observed (28.0%). This is probably due to its hygroscopicity. The quantitative ORAC and TPC also showed a significant decrease in the ExMR AOA (from 2.6 to 0.7 ORAC units) and TPC (about 12.0%) (Table 3). Moreover, while CDExMR values slightly decreased (about 5.0%), ExMR2 and ALGCDExMR remained quite unaltered, showing that this significant result in terms of stability (<1%) is due to ALG that is able to enhance ExMR *shelf life.*

The decrease of ExMR data was probably due to thermal degradation of few polyphenols contained in citrus extract. In fact, literature data report that anthocyanins in blood orange juice presented high-rate constant of degradation in the range 30°–90°C [49] with 69% losses after 90° for 120 min [50]. Instead, for flavanones such as hesperidin, no significant decrease was noticed in the range 70–90°C after 240 min [51]. In fact, it was demonstrated that glycosidic flavonoids are more resistant than aglycone flavonoids to heat treatments [52].

### 3.6. Antioxidant Efficiency

The ORAC assay has been performed in order to evaluate the qualitative antioxidant efficiency of the best formulation, untreated and treated according to ICH. The data (Figure 1) showed that at 4 hours, ALG and CD slightly improve the ExMR antioxidant efficiency with respect to the unformulated extract, with a major effect for CD polymer. On the contrary, at 6 hours, ExMR and all the formulations without CD (ExMR2 and ExMR ICH) had lost their antioxidant efficiency. Indeed, ALG/CD/ExMR and ALG/CD/ExMR ICH maintain and prolong the extract activity only with a slow further spontaneous decomposition of fluorescein. These results suggest that the presence of CD in the microsystems preserved the antioxidant efficiency of the extract for a longer time and enhanced the stability of the extract.

### 3.7. Antiglycation Activity

**Figure 7:**
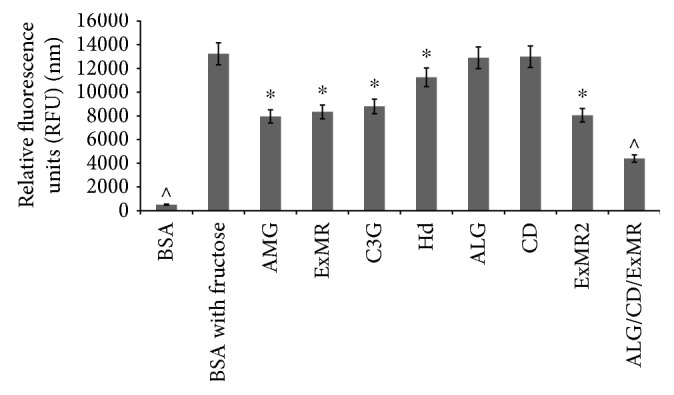
Antiglycation effect of unformulated and formulated extracts, Hd and C3G, major biopolyphenol compounds, in comparison with AMG assay standard control. Results are means ± SD. Significantly different at ^∗^*p* < 0.05 or ^^^*p* < 0.01 compared with positive control (BSA with fructose).

The amount of AGEs is elevated during hyperglycemic and/or oxidative stress conditions [53]. This process induces irreversibly fluorescent macroprotein derivative formation, termed AGEs, via Maillard reaction [54]. Considering that a diet rich in natural antioxidant compounds protect against protein glycation [18], we evaluated the inhibitory effect of both unformulated and formulated extracts on fluorescent AGE formation. The results (Figure 7) showed a good ExMR capability to inhibit AGEs, reducing the max fluorescent value of positive control (BSA with fructose) from 13,254 nm to 8338 nm. This value corresponded to 37.0% of inhibition, and the observed effect is superimposable to that of the AMG assay standard (40.0% of inhibition). Also, in order to determine which representative compounds influence the most the antiglycation effect, the ExMR activity was compared with those of C3G and Hd, the selected representative standard compounds. Both samples are able to inhibit AGE formation, showing a fluorescence of 8798 nm and 11,249 nm, respectively, corresponding to 36.3% and 15.2% of inhibition. This demonstrates that the ExMR activity is due to a synergic effect of both polyphenol class compounds (bioflavonoids and anthocyanins). Moreover, to evaluate the polymer effect, ExMR2 and ALGCDExMR were also tested. The major activity of formulation was found in ALGCDExMR with a fluorescence value of 4335 nm, corresponding to 69.2% of inhibition, followed by ExMR2 with a fluorescence value of 8040 nm, corresponding to 39.3% of inhibition. The results underline that all the formulations tested showed an AGE direct inhibition, indicating that both CD and ALG polymers are able to protect the extract from degradation and oxidation phenomena.

### 3.8. Inhibitory Activity on MMP-2 and MMP-9

AGE accumulation has a role in the increase of different metalloproteinase expression [18]. Because many polyphenol compounds inhibit both collagenase and gelatinase activities [55, 56], we evaluated the inhibition ability of the tested samples on MMP-2 and MMP-9.

The results reported in Table 4, expressed as the concentration value (*μ*g/ml) of potential inhibitor that reduces of 50% the MMP activity (IC_50_), showed that ExMR possesses a high capability to inhibit both MMP-2 and MMP-9. This could be due to the anthocyanin content. In fact, the representative flavonoid Hd is not active, while the anthocyanin C3G has an IC_50_ value of 10.57 ± 1.35 *μ*g/ml and 7.27 ± 1.05 *μ*g/ml on MMP-2 and MMP-9, respectively.

In order to evaluate if the formulation can influence the activity of the pure extract, the inhibitory activity of formulated extract (ExMR2 and ALGCDExMR) and pure materials (ExMR, ALG and CD) were assayed. We observed that the presence of ALG in ExMR2 reduced the inhibition activity on MMP-2 (4.49 ± 0.85 *μ*g/ml), but improved the activity on MMP-9 (4.27 ± 0.46 *μ*g/ml), while the presence of CD in ALGCDExMR improved the inhibitory effect on both MMPs (0.49 ± 0.09 *μ*g/ml and 1.40 ± 0.19 *μ*g/ml on MMP-2 and MMP-9, resp.) with respect to pure ExMR (1.12 ± 0.12 *μ*g/ml and 5.52 ± 0.72 *μ*g/ml, resp.). This could be explained with a high inhibition effect of CD on MMP-2 (4.03 ± 0.35 *μ*g/ml) and MMP-9 (2.98 ± 0.31 *μ*g/ml), probably due to the presence of free hydroxyl groups that support the hydrogen bond with the enzyme-active site [57].

## 4. Conclusions

An easily spray-dried handle antioxidant extract (ExMR) was produced to formulate dietary supplements for human health as well-formed and stable microparticles of ALG and CD also to make it suitable to be added in baked goods as bioactive food ingredients. The bioactivity on dysmetabolic disease was due to extract polyphenol compounds. In fact, anthocyanins and bioflavonoids both resulted responsible for the dried extract in vitro AGE inhibition activity, while only the anthocyanin content was effective with respect to in vitro MMP inhibition. Also, CD and ALG polymers were able to improve these activities. On one hand, CD improved the MMP inhibitory activity of the extract presumably because of the presence of free hydroxyl groups that support the hydrogen bond with the enzyme-active site; on the other hand, CD acted synergistically with ALG enhancing the dissolution/release of ExMR and improving the in vitro AGE direct inhibition of extract, also protecting it from degradation and oxidation phenomena. Furthermore, CD preserved the extract antioxidant efficiency and stability, while the choice of 2% of ALG as coated polymer (3 : 1 ALG/ExMR weight ratio) was effective to improve the extract wettability and its *shelf life*.

This research represents an advantageous way to re-evaluate a blood orange citrus by-products of the Sicilian industry and to develop human dietary supplement which also acts to be added as “bioactive food ingredients” in functional foods like baked goods.

## Supplementary Material

Graphical abstract. ExMR spray-dried extract, rich in polyphenol compounds, was obtained from a blood orange fluid extract. ExMR, properly formulated with an alginate/cyclodextrin matrix, give water soluble microsystems highly capable to act on dysmetabolic diseases.

## Figures and Tables

**Table 1:** Total anthocyanin content of ExMR and ExF and relative percentage of individual anthocyanins.

Peak number^a^	*t*_R_ (min)	(*M*)^+^ (m/z)	MS*^n^* (m/z)	Anthocyanins	Relative composition^b^ (%)	
					ExMR	ExF
1	13.9	611	449/287	Cyanidin 3,5-diglucoside	1.31 ± 0.01	1.72 ± 0.08
2	14.2	465	303	Delphinidin 3-glucoside	2.65 ± 0.02	2.96 ± 0.13
3	14.5	611	287	Cyanidin 3-sophoroside	0.51 ± 0.01	0.16 ± 0.02
4	15.2	449	287	Cyanidin 3-glucoside	39.87 ± 0.17	31.80 ± 0.06
5	15.9	595	287	Cyanidin 3-rutinoside	1.34 ± 0.24	0.43 ± 0.10
6	16.8	479	317	Petunidin 3-glucoside	1.55 ± 0.02	1.53 ± 0.02
7	17.5	551	465/303	Delphinidin 3-(6″-malonyl)glucoside	1.53 ± 0.01	0.67 ± 0.08
8	17.9	463	301	Peonidin 3-glucoside	2.88 ± 0.03	2.90 ± 0.03
9	18.2	565	479/317	Petunidin 3-(6″-malonyl)glucoside	1.48 ± 0.01	0.58 ± 0.05
10	18.9	535	449/287	Cyanidin 3-(6″-malonyl)glucoside	21.73 ± 0.10	36.40 ± 0.05
11	19.9	593	449/287	Cyanidin 3-(6″-dioxalyl)glucoside	5.91 ± 0.03	8.02 ± 0.05
12	20.4	—	271	Pelargonidin derivative	1.23 ± 0.01	5.55 ± 0.03
13	21.7	549	463/301	peonidin 3-(6″-malonyl)glucoside	13.84 ± 0.11	5.81 ± 0.12
14	22.2	—	287	Cyanidin derivative	2.35 ± 0.03	1.01 ± 0.12
15	23.3	—	301	Peonidin derivative	1.82 ± 0.02	0.46 ± 0.08
Total anthocyanins (g C3G/100 g extract)^c^					0.81 ± 0.01	0.32 ± 0.02

^a^The numbering is according to Figures 2(a) and 2(b). ^b^Relative percentage of anthocyanins was based on HPLC peak areas recorded at 520 nm. ^c^Anthocyanins are expressed as cyanidin 3-glucoside equivalents.

**Table 2:** Concentration of individual flavanones and total flavanones in ExMR and ExF.

Peak number^a^	*t*_R_ (min)	(*M*–*H*)^−^ (m/z)	MS*^n^* (m/z)	Flavanones	g Hd/100 g^b^	
					ExMR	ExF
1	13.5	579	271	Narirutin	5.48 ± 0.05	2.22 ± 0.06
2	18.5	609	301	Hesperidin	5.73 ± 0.02	2.35 ± 0.11
3	59.9	593	285	Didymin	0.79 ± 0.01	0.33 ± 0.02
Total flavanones (g Hd/100 g extract)^b^					12.00 ± 0.07	4.90 ± 1.13

^a^The numbering is according to Figures 2(c) and 2(d). ^b^Flavanones are expressed as hesperidin equivalents.

**Table 3:** Quantitative antioxidant activity (ORAC assay) and total polyphenol content (TPC) of fluid extract (ExF), spray-dried extract (ExMR), and spray-dried extract processed according to ICH guidelines (ExMR ICH).

Sample	TPC (mg GAE/g of extract**)**	ORAC units (*μ*mol TE/*μ*g of extract)
ExF	12.00 ± 0.18	1.90 ± 0.80^∗^
ExMR	33.50 ± 0.21	2.60 ± 0.30^∗^
ExMR ICH	28.80 ± 0.60	0.70 ± 0.08^∗^

Reported values are the means ± standard deviation (SD) (*n* = 3). ^∗^Significantly different at *p* < 0.05 compared to 1 ORAC unit of Trolox.

**Table 4:** Inhibitory activity on MMP-2 and MMP-9.

Compounds	IC_50_ MMP-2 (*μ*g/ml)	IC_50_ MMP-9 (*μ*g/ml)
ExMR	1.12 ± 0.12	5.52 ± 0.72
ALG	n.a.	n.a.
CD	4.03 ± 0.35^^^	2.98 ± 0.31^∗^
Hd	n.a.	n.a.
C3G	10.57 ± 1.35^^^	7.27 ± 1.05
ExMR2	4.49 ± 0.85^^^	4.27 ± 0.46^∗^
ALGCDExMR	0.49 ± 0.09^^^	1.40 ± 0.19^^^

n.a. = not active (IC_50_ > 100 *μ*g/ml). Reported values are the means ± standard deviation (SD) (*n* = 3). ^∗^*p* < 0.05 compared to ExMR value. ^^^*p* < 0.01 compared to ExMR value.
