# Supplementary material for: An Alginate/Cyclodextrin Spray Drying Matrix to Improve Shelf Life and Antioxidant Efficiency of a Blood Orange By-Product Extract Rich in Polyphenols: MMPs Inhibition and Antiglycation Activity in Dysmetabolic Diseases
Source: Oxid Med Cell Longev. 2017 Nov 2;2017:2867630. doi: 10.1155/2017/2867630 (PMC5688344; doi:10.1155/2017/2867630)
Supplement: Supplementary file 1 — Graphical abstract. ExMR spray-dried extract, rich in polyphenol compounds, was obtained from a blood orange fluid extract. ExMR, properly formulated with an alginate/cyclodextrin matrix, give water soluble microsystems highly capable to act on dysmetabolic diseases. [file 2867630.f1.pptx]

## Slide 1
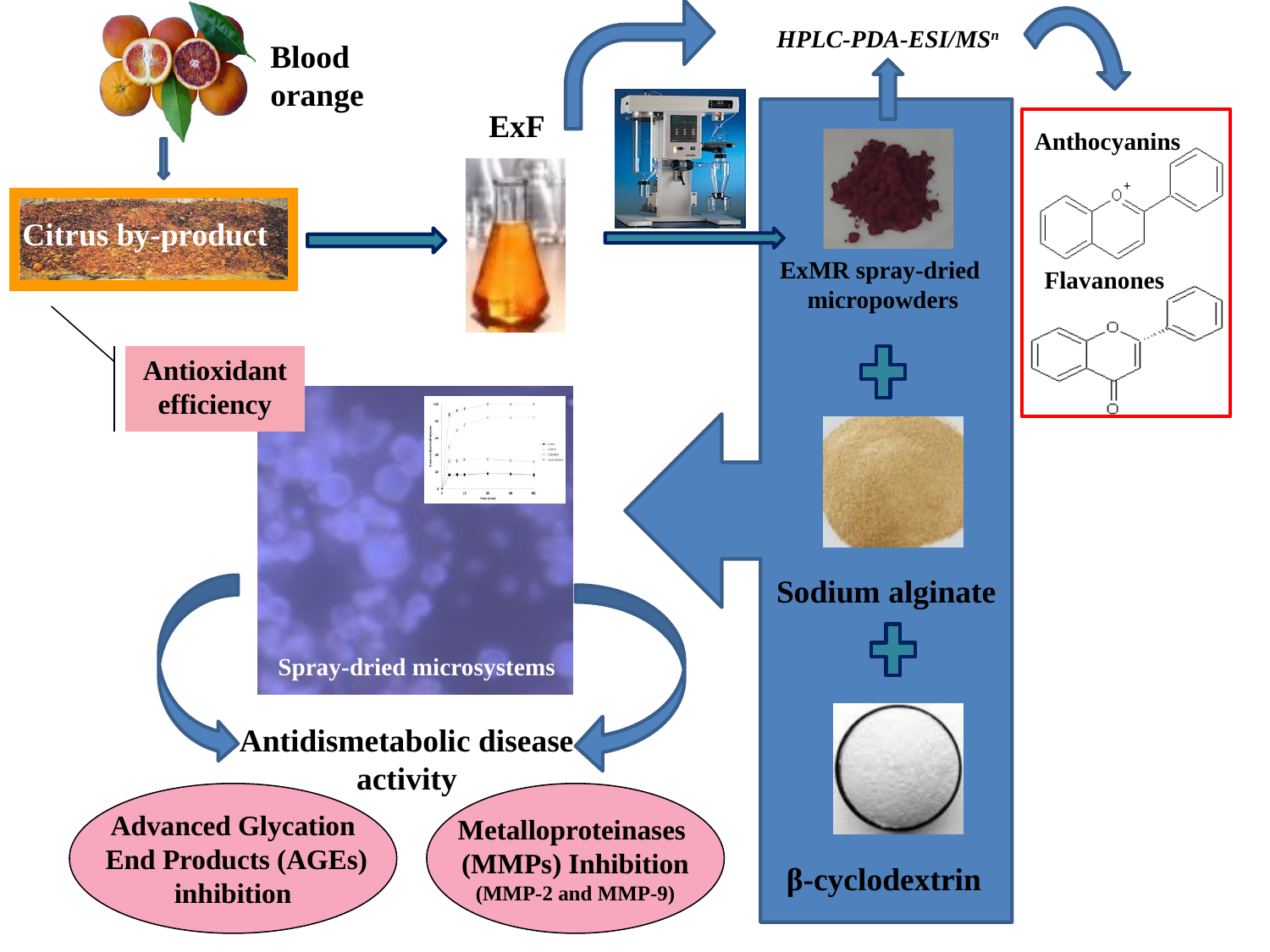

HPLC-PDA-ESI/MSn
Blood orange
ExF
Anthocyanins
Citrus by-product
ExMR spray-dried micropowders
Flavanones
Antioxidant efficiency
Sodium alginate
Spray-dried microsystems
Antidismetabolic disease activity
Advanced Glycation
 End Products (AGEs)
inhibition
Metalloproteinases
(MMPs) Inhibition
(MMP-2 and MMP-9)
β-cyclodextrin
